# Anchoring links in Chinese pre-service teachers’ role beliefs about typically developing students and those with intellectual and developmental disabilities: a metaphorical analysis

**DOI:** 10.3389/fpsyg.2026.1808614

**Published:** 2026-06-04

**Authors:** Weihao Xin, Xian Wang, Zhenzhen Zhang, Lingmei Cao

**Affiliations:** 1 Jing Hengyi School of Education, Hangzhou Normal University, Hangzhou, China; 2 Zhejiang Philosophy and Social Science Laboratory for Research in Early Development and Childcare, Hangzhou Normal University, Hangzhou, China; 3 Department of Special Education, Faculty of Education, East China Normal University, Shanghai, China; 4 School of Languages and Cultural Communication, Shanghai University of Finance and Economics Zhejiang College, Jinhua, China

**Keywords:** belief systems, China, inclusive education, intellectual and developmental disabilities, metaphor analysis, pre-service teachers, teacher role beliefs

## Abstract

**Introduction:**

Teachers’ beliefs are central to shaping inclusive practices, yet evidence remains limited on how pre-service teachers articulate role beliefs in parallel for typically developing students and for students with intellectual and developmental disabilities (IDD), and on how these belief patterns relate within a broader belief system.

**Methods:**

This study investigated Chinese pre-service teachers’ role beliefs through metaphor analysis, using a sample of 356 participants.

**Results:**

Protective orientations predominated in both student groups. Beyond this shared emphasis, metaphors for typically developing students were more frequently coded as behavioral, whereas metaphors for students with IDD showed relatively greater representation of self-centered and facilitative orientations. Academic year and teacher role beliefs for typically developing students were significantly associated with those for students with IDD.

**Discussion:**

By highlighting similarities and differences between these belief patterns and examining their cross-target associations, this study contributes to understanding how pre-service teachers articulate role beliefs across student groups and identifies potential directions for teacher education to strengthen inclusive practice.

## Introduction

As Pajares (1992) noted, “all teachers hold beliefs about their work, their students, their subject matter, and their roles and responsibilities” (p. 314). For pre-service teachers, these beliefs, defined as “psychologically held understandings, premises, or propositions about the world that are perceived as true” (Richardson, 2003, *p*. 2), serve as cognitive filters that shape how they interpret new information, organize knowledge, and approach teaching (Fives and Buehl, 2012), with particular consequences for learning to teach in inclusive and special education contexts. Such beliefs influence not only teachers’ attitudes toward students with intellectual and developmental disabilities (IDD) and inclusive education, but also core instructional processes—including the planning, delivery, and adaptation of instruction—to address students’ diverse needs (Jordan, 2018). Accordingly, Sharma (2018) described these beliefs as “the heart of inclusive teachers,” emphasizing that cultivating them is essential to preparing educators to create high-quality, equitable, and inclusive learning environments.

Among teachers’ beliefs, teacher role beliefs—which concern their understandings of professional responsibilities, role boundaries, and obligations to act—are particularly important (Xin et al., 2020, 2024a, 2026a). Fives and Buehl (2012) conceptualize teachers’ beliefs as an umbrella term encompassing broad beliefs about teaching and learning, including the purposes of education, how students learn, appropriate pedagogy, classroom management, and teacher–student relationships. Within this broader belief system, teacher role beliefs constitute a more specific facet that foregrounds teachers’ perceived responsibilities, role boundaries, and obligations to act (Domović et al., 2017). In other words, they concern what teachers believe they should do and be accountable for when teaching particular student groups. Accordingly, role beliefs are best viewed as a domain-specific subset of teachers’ broader belief systems rather than an entirely separate construct. By shaping how responsibility is attributed (e.g., to the teacher, the student, or the wider support system), these beliefs influence teachers’ perceived control over learning processes and their interaction patterns with diverse learners (Li et al., 2024). Over time, role beliefs also contribute to teachers’ role identity, reflecting a professional self-understanding that develops throughout their careers (Alaklabi and Low, 2022). Examining pre-service teachers’ role beliefs, therefore, offers insight into who they aspire to be as teachers, how they wish to be perceived, and how they conceptualize and enact professional responsibilities—issues that are especially salient in inclusive and special education teacher preparation (Kokkosi et al., 2021). Neglecting these beliefs may lead teacher educators to overlook default role scripts and assumptions about responsibility that shape future teachers’ effectiveness in diverse classrooms (Domović et al., 2017).

Despite the recognized importance of teachers’ role beliefs, scholarship in this domain remains limited compared with the extensive literature on teachers’ broader beliefs about teaching and learning (Alaklabi and Low, 2022), and studies specifically examining pre-service teachers’ role beliefs are even scarcer. Existing research on pre-service teachers’ role beliefs for typically developing students (i.e., students without identified disabilities in general education settings) and for students with IDD often (a) aggregates disability categories, thereby overlooking subgroup-specific role expectations, and (b) rarely examines role beliefs for typically developing students and students with IDD in parallel within a shared analytic framework (Domović et al., 2017). Students with IDD constitute a substantial portion of the diverse learner population in China and often exhibit distinctive and wide-ranging cognitive, behavioral, and social–emotional needs (Xin et al., 2024a,b). These characteristics may challenge teachers and are likely to shape expectations, instructional strategies, and perceived professional responsibilities in ways that differ from those for typically developing students (Jordan, 2018). Consequently, it is essential to understand how pre-service teachers conceptualize their professional roles in relation to students with IDD. However, it remains unclear whether such role beliefs differ from, or relate to, those for typically developing students, and how they may be related within broader teacher belief systems. To address this gap, the present study examines the relationship between pre-service teachers’ role beliefs for typically developing students and for students with IDD, thereby clarifying patterned similarities, differences, and associations in these beliefs and identifying actionable entry points for teacher education to strengthen inclusive practices.

### Literature review

#### Teachers’ role beliefs and metaphorical reasoning

Educational reforms over the past few decades have redefined expectations for both teachers and students, fostering meaningful learning (Ahonen et al., 2014). Whether these reforms translate into practice depends, in part, on how teachers understand their professional roles and responsibilities in relation to student learning (Lloyd, 2005). Teachers’ role beliefs capture these understandings and guide how they interpret responsibilities, engage with students, and make instructional decisions (Xin et al., 2020,2024a, 2026a). For pre-service teachers, whose professional identities are still forming, early role beliefs may become durable frames that shape later classroom practice (Fives and Buehl, 2012). Accordingly, clarifying the content and patterning of pre-service teachers’ role beliefs is foundational to understanding how teachers come to enact particular patterns of instruction, support, and responsibility in diverse classrooms, including how these role beliefs may be recalibrated when teachers imagine working with different student groups.

Because teachers’ role beliefs are partly implicit and not always readily verbalized, the methods used to study them also shape what can be learned about how these beliefs are expressed, organized, and made available for interpretation (Pajares, 1992). Research on pre-service teachers’ role beliefs has typically relied on semi-structured interviews, which can illuminate the nuanced and sometimes contradictory nature of these conceptualizations (Gao and Cui, 2024). However, role beliefs often include tacit, difficult-to-articulate elements that may be only partially captured through direct questioning (Fives and Buehl, 2012). Metaphor analysis is well suited to this challenge because metaphors are not merely decorative linguistic expressions but cognitive and conceptual resources through which individuals organize, interpret, and communicate abstract experience (Domović et al., 2017). Conceptual metaphor theory holds that people often understand abstract target domains through more concrete or familiar source domains, so metaphorical expressions can illuminate the conceptual mappings that participants draw on when articulating their views, rather than isolated word choices (Lakoff and Johnson, 1980). From this perspective, when a pre-service teacher describes a teacher as a “gardener,” “lighthouse,” or “parent,” the metaphor does not simply provide a vivid image; it offers a window into how the teacher role is construed in that response, including assumptions about growth, guidance, protection, responsibility, agency, and teacher-student relations.

Psychological accounts of metaphorical reasoning further support this interpretation. Structure-mapping theory holds that metaphor comprehension involves aligning relational structures between a source and a target domain, thereby enabling the projection of selected relations from a familiar domain onto a less clearly articulated one (Gentner, 1983). Related work on metaphor processing also indicates that metaphors can highlight certain aspects of a target concept while downplaying others, thereby shaping how people reason about social roles, responsibilities, and possible actions (Bowdle and Gentner, 2005). Applied to teacher role beliefs, metaphors can therefore make visible the relational and evaluative structures through which pre-service teachers imagine what teachers should do, what students need, and how teaching should be enacted in particular contexts (Xin et al., 2026a,b,c). This theoretical grounding justifies using metaphors as meaningful analytic access points to how teachers’ role beliefs are articulated and patterned, especially when those beliefs are implicit, emerging, or only partially available through direct self-report.

Accordingly, metaphor analysis has increasingly been used to examine how pre-service teachers construe professional roles and identities (Kokkosi et al., 2021). In this approach, participants are invited to express the teacher role through a metaphor (e.g., “A teacher is like …”) and to provide a brief explanation for their choice. Researchers then analyze the metaphorical expression alongside its justification to interpret the role logic expressed in the response (Domović et al., 2017). Analytically, the procedure typically involves (a) identifying the metaphorical vehicle (such as “lighthouse” or “gardener”), (b) interpreting the intended mapping based on the accompanying explanation, and (c) coding each response into theoretically defined categories using an explicit coding protocol to ensure comparability across participants (Xin et al., 2020). The accompanying explanation is particularly important because the same metaphorical vehicle may imply different mappings across participants. For example, “gardener” may emphasize patient cultivation, behavioral pruning, or protective care, depending on how participants justify the comparison. Analyzing both the vehicle and its explanation, therefore, reduces the risk of treating metaphors as fixed labels and allows the researcher to identify the role logic that the participant assigns to the metaphor. By eliciting non-literal mappings, metaphor analysis can surface implicit role logics that complement interview-based approaches and support systematic comparison of role beliefs across student groups (Ulusoy, 2022).

Given that metaphors can reflect different pedagogical logics, it is important to specify the metaphor-based categories before presenting empirical findings. Metaphor-based research in this area commonly organizes role beliefs into four orientations that reflect distinct role logics (Alaklabi and Low, 2022; Domović et al., 2017): (a) self-centered orientations emphasize teachers’ self-focused concerns about performance and adequacy; (b) behavioral orientations foreground transmission, authority, and control; (c) protective orientations prioritize care and emotional safety; and (d) facilitative orientations highlight guidance, autonomy support, and scaffolded learning. Importantly, this four-orientation framework provides analytically useful categories that can be operationalized through protocol analysis, enabling systematic coding and comparison of role beliefs across student groups. In the present study, these orientations are treated as interpretive categories derived from participants’ metaphorical responses and accompanying explanations, rather than as direct measures of stable internal cognitive structures. Accordingly, the present study uses this four-orientation framework as an analytic tool to systematically and comparably interpret metaphors for both typically developing students and students with IDD.

#### Pre-service teachers’ role beliefs for typically developing students and students with IDD

Findings from metaphor- and interview-based studies converge on the view that pre-service teachers’ role beliefs are not fixed but can shift as teachers progress through training and as the imagined teaching context changes (Fives and Buehl, 2012). Longitudinal evidence suggests that teachers’ role conceptualizations can shift during teacher education, sometimes moving from teacher-centered authority metaphors toward more facilitative ones (e.g., from “leader” to “lighthouse”; Vidović and Domović, 2019; Niikko, 2022). At the same time, the persistence of teacher-centered and behaviorist metaphors has also been documented, indicating that change is neither universal nor guaranteed (Ulusoy, 2022). Cross-sectional findings further show that teachers’ role beliefs are context-sensitive and may vary with the student populations teachers imagine or encounter. For instance, Chinese STEM-oriented pre-service teachers were more likely to endorse facilitative roles with migrant students (Wang and Yang, 2021), whereas Greek preschool teachers’ metaphors ranged from knowledge transmitters to collaborators and caregivers (Kokkosi et al., 2021). Taken together, this literature suggests that student-group characteristics may serve as salient cues that activate distinct role scripts, underscoring the importance of examining role beliefs across specific student populations—including comparisons between typically developing students and those with IDD.

This sensitivity to student groups is particularly salient in inclusive and special education contexts, where teachers may face distinct expectations and responsibilities (Jordan, 2018) and where variations in support intensity can make role boundaries and assumptions about responsibility more visible. Consistent with the context-dependent nature of teachers’ role beliefs, a growing body of research has examined these beliefs in relation to students with disabilities (Xin et al., 2020, 2024a, 2026a). Empirically, Croatian general education pre-service teachers tended to endorse self-centered or facilitative orientations, with behavioral orientations least common (Domović et al., 2017). Similarly, Chinese special education pre-service teachers showed stronger facilitative orientations, particularly when they believed students could develop through appropriate interventions (Xin et al., 2020). Evidence from in-service samples suggests that these orientations may persist into professional practice (Alaklabi and Low, 2022; Orozco and Moriña, 2023), underscoring the importance of the pre-service stage in shaping role beliefs relevant to inclusive teaching.

Nevertheless, a key limitation of the disability-focused literature is its coarse analytical granularity. Many studies treat “students with disabilities” as a single group, which can obscure subgroup-specific role expectations, including those relevant to students with intellectual and developmental disabilities (IDD; Domović et al., 2017). This distinction matters because students with IDD often require pervasive, long-term supports spanning learning, communication, behavior regulation, and adaptive functioning (Xin et al., 2024a). These support demands are likely to elicit systematically different expectations for teachers’ roles, particularly regarding the distribution of responsibility among the teacher, the student, and the broader support system (Xin et al., 2024b), and may therefore be reflected in distinct orientation profiles within a shared coding framework. Accordingly, collapsing disability groups into one category risks masking role-belief patterns that may be distinctive to IDD.

Beyond the issue of subgroup specificity, the literature rarely examines role beliefs for typically developing students and those in disability-related contexts side by side within a shared analytic framework (Domović et al., 2017). As a result, the similarities and differences between pre-service teachers’ role beliefs for typically developing students and those for students with IDD remain insufficiently understood. This gap is not merely descriptive. Without an explicit comparison across student groups, it is difficult to distinguish between role-belief orientations that function as relatively stable, generalized role scripts and those that reflect contingent adaptations to different student populations. Consequently, teacher education may lack an evidence-based basis for setting intervention priorities. For example, programs may assume that facilitative beliefs articulated for typically developing students will automatically generalize to students with IDD, or, conversely, may overextend disability-related findings to mainstream classroom contexts, potentially resulting in misaligned training emphases and uneven learning opportunities for students with IDD.

These gaps are particularly consequential in pre-service teacher education, where role beliefs are still forming and may become durable guides for later practice. Yet research directly examining pre-service teachers’ role beliefs toward students with IDD remains scarce. Existing evidence indicates that role beliefs shape teachers’ instructional priorities and their willingness to assume responsibility for diverse learners (Jordan, 2018). Pre-service teachers may enter training with assumptions that do not align with the practical demands of inclusive classrooms (Gavish, 2017; Domović et al., 2017). Demands such as balancing individualized supports with whole-class instruction and collaborating with families may further complicate the allocation and enactment of responsibility, revealing gaps in preparedness. Accordingly, examining how pre-service teachers construe their roles with students with IDD and how these beliefs relate to their role beliefs for typically developing students can clarify the patterned relations among role beliefs and inform teacher education aimed at preparing educators for diverse learners. Building on evidence that role beliefs are both context-sensitive and systematically classifiable, the present study applies the four-orientation framework to (a) compare orientation distributions across the two student groups and (b) test whether role beliefs for typically developing students are associated with, and may provide an anchoring frame for, role beliefs for students with IDD.

### Theoretical framework

Rokeach’s (1968) Theory of Belief Systems provides a useful framework for understanding pre-service teachers’ professional beliefs. Beliefs are organized hierarchically, with central beliefs—formed early, highly interconnected, and resistant to change—constituting the core of the system, while peripheral beliefs—developed later, less connected, and more flexible—are more open to modification (Gao and Cui, 2024; Pajares, 1992). In this framework, centrality refers to the degree to which a belief is psychologically embedded within the broader belief system (Rokeach, 1968). Central beliefs underpin peripheral ones, shaping how they are interpreted, prioritized, and integrated into practice (Richardson, 2003).

Within teachers’ belief systems, teacher role beliefs may be relatively salient or influential, helping to organize professional interpretations and role expectations, shaping and constraining more peripheral professional beliefs, such as those about instructional practice and classroom management, and influencing professional thinking, including perceptions of student potential (Fives and Buehl, 2012). This hierarchical view is useful for the present study because it allows teacher role beliefs to be examined not only as isolated orientations but also as potentially related elements within a broader professional belief system. However, the present study does not directly measure belief centrality as a psychological property. Specifically, it does not assess the degree to which a belief is connected to other beliefs, resistant to change, or embedded in the self-system. Therefore, Rokeach’s distinction between relatively central and relatively peripheral beliefs is used here as a theoretical lens for formulating an anchoring hypothesis, rather than as an empirically established classification assumed in advance.

Building on this theoretical distinction, the present study examines whether role beliefs formed in relation to typically developing students may serve as reference points when pre-service teachers construe their roles with students with IDD. In the context of inclusive and special teacher education, pre-service teachers’ role beliefs regarding typically developing students may serve as relatively established reference frames (Alaklabi and Low, 2022). Formed early through personal schooling experiences, cultural norms, and exposure to role models, these beliefs tend to be more familiar and more frequently activated, and may guide expectations, instructional decisions, and the development of other professional beliefs (Domović et al., 2017). Teachers’ role beliefs for students with IDD, in contrast, often develop later and may therefore be less consolidated and more context-sensitive, particularly when pre-service teachers have limited direct experience with students with IDD (Xin et al., 2020). Importantly, centrality should be understood as contingent and potentially malleable. With sustained and meaningful experience teaching students with IDD, IDD-related role beliefs may be repeatedly activated and reinforced, becoming more psychologically embedded and, for some individuals, more central within the broader belief system (Xin et al., 2024a). Accordingly, rather than assuming that role beliefs for typically developing students are definitively central and those for students with IDD are definitively peripheral, the present study treats this relationship as a theoretically grounded proposition to be examined empirically. Specifically, we examine whether role beliefs for typically developing students are systematically associated with those for students with IDD, in ways that may be compatible with an anchoring interpretation.

However, Rokeach’s (1968) Theory of Belief Systems is not the only possible interpretation of cross-target role-belief associations. From a teacher socialization perspective, similarities in role beliefs for typically developing students and those with IDD may reflect shared institutional norms, cultural expectations of teaching, and common curricular experiences rather than hierarchical belief centrality *per se* (Zeichner and Gore, 1990). From a professional identity perspective, the observed associations may reflect pre-service teachers’ emerging sense of the kind of teacher they wish to become across different instructional contexts (Beauchamp and Thomas, 2009). From a program-exposure perspective, disability-related coursework, practicum opportunities, and contact with students with disabilities may shape how role beliefs are transferred, revised, or reconfigured (Forlin et al., 2009). These alternative interpretations are not mutually exclusive with Rokeach’s framework. Rather, they indicate that the present study should be understood as testing whether the observed patterns are consistent with an anchoring process, while recognizing that such patterns may also be shaped by socialization, identity development, and program exposure.

This more cautious theoretical positioning also clarifies the present study’s empirical contribution. Despite the theoretical emphasis on this anchoring process in Rokeach’s (1968) Theory of Belief Systems, few studies have examined how pre-service teachers’ more established role beliefs for typically developing students relate to, and potentially frame, role beliefs for students with IDD. Early research has either addressed teacher belief systems broadly, examining relations among beliefs about students, self-as-teachers, and teaching practices, or lacked a theoretical perspective on the hierarchical organization of beliefs, often treating teachers’ role beliefs for typically developing students separately from those concerning students with disabilities. Such separation can obscure how pre-existing role beliefs about typically developing students may anchor or interact with the development of beliefs about more diverse learners, limiting our understanding of how inclusive teaching orientations emerge. By assessing role beliefs for both student groups using the same orientation framework and testing their cross-target associations, the present study provides an empirical entry point for examining whether the observed pattern is compatible with this theorized anchoring process. Importantly, this study does not claim to prove the centrality of one set of role beliefs or the peripheral status of another. Instead, it tests whether the association between the two sets of role beliefs is consistent with the anchoring proposition derived from Rokeach’s (1968) Theory of Belief Systems, while remaining open to alternative interpretations.

### The Chinese context

Special education in China initially served students who were blind or deaf and was later extended to those with IDD (Yu et al., 2011). Today, students with IDD are educated in both segregated special schools and inclusive general schools, necessitating teachers’ ability to work effectively across diverse settings and to negotiate role expectations across contexts (Xin et al., 2025). However, traditional teacher preparation pathways were narrowly focused. Special education programs primarily trained teachers for segregated special schools, emphasizing courses in special education theory, practice, and pedagogy for students with IDD, while providing minimal subject-specific or general education preparation (Liu, 2021). In contrast, general education programs primarily train teachers for typically developing students, focusing on general education theory and practice and instruction in specific subjects or educational stages, while offering minimal exposure to special education or inclusive practices (Fu, 2022). Consequently, when special education graduates take on roles as resource or itinerant teachers, and general education graduates take on roles as inclusive classroom teachers, both groups may be underprepared in at least one key domain—either the disciplinary expertise or the instructional skills needed to support students with IDD in inclusive settings (Liu, 2021).

Over the past 15 years, Chinese national policies have broadened the scope of special education to include all students with disabilities, establishing the expectation that special education teacher candidates can work effectively in both segregated special schools and inclusive schools, where students with IDD are increasingly enrolled (Yu et al., 2011). These policies have defined the preparation and professional requirements for pre-service special education teachers, emphasizing the development of “compound-competency” teachers. Such teachers combine special education expertise with additional qualifications (“Special Education + X”), where X may refer to a subject area (e.g., Chinese, mathematics), an educational stage (e.g., early childhood, primary), or rehabilitation skills such as speech, physical, or occupational therapy (Liu, 2021). Initiatives such as the 2014 and 2018 Excellence Teacher Training Programs have promoted integrated preparation models that explicitly pair special education training with subject-specific or rehabilitation instruction. By redefining what future special education teachers are expected to do across settings, these reforms also provide a consequential context in which role beliefs may be formed and negotiated. These models guide future special education teachers’ professional development and role readiness and are intended to strengthen their capacity to support students with IDD across settings.

Inclusive education reforms have further emphasized the need for all general education teachers to acquire foundational special education knowledge and skills to work effectively with students with IDD (Yu et al., 2011). Recent strategies, including the successive Special Education Promotion Plans (2014–2016; 2017–2020; 2021–2025), require embedding special education content in general education teacher qualification examinations and pre-service curricula. Under the proposed tiered model, all teacher candidates complete compulsory inclusive education foundation courses. Those seeking more specialized expertise can choose elective modular courses in special education, particularly at institutions with established programs. These courses are designed to enhance teachers’ capacity to support students with IDD in inclusive education settings (Wang, 2023). Together, these policy moves increase the likelihood that general education pre-service teachers will develop role beliefs about students with IDD, raising questions about how these beliefs relate to their more established role beliefs about typically developing students.

Collectively, these reforms aim to prepare a teaching workforce capable of meeting the diverse needs of all students, including those with IDD, across both special and inclusive education contexts in China (Fu, 2022). International evidence suggests that the success of educational reforms depends on shifts in teachers’ roles (Ahonen et al., 2014), particularly their readiness to support diverse learners, including students with IDD. However, little is known about how Chinese pre-service teachers conceptualize their professional roles for typically developing students and for students with IDD within their broader belief systems, including whether these belief patterns align or are compatible with anchoring. Understanding these beliefs is critical because teacher preparation programs shape role perceptions that guide responsiveness to learner diversity. These perceptions also underpin pre-service teachers’ readiness to enact roles such as inclusive classroom teachers, resource teachers, or itinerant teachers—a priority in China’s ongoing reforms to strengthen teacher preparation for educating students with IDD (Wang, 2023).

### Present study

This study examines how Chinese pre-service teachers conceptualize their professional roles with typically developing students and with students with IDD, and whether the association between these two sets of role beliefs aligns with a theoretically proposed anchoring process. Investigating teachers’ role beliefs across these groups, identifying their similarities and differences, and analyzing their interconnections offer two complementary contributions, namely a parallel framework for consistent comparison across student groups and an empirical examination of cross-target role-belief associations within a belief-system perspective. This knowledge can inform teacher education initiatives that foster diverse and inclusive role beliefs from the outset, supporting pre-service teachers in developing flexible, context-sensitive professional identities that promote inclusive classroom practices. Using metaphor analysis coded with the four-orientation framework, this study addresses the following research questions (RQs):

1. What are the similarities and differences between pre-service teachers’ role beliefs about typically developing students and those about students with IDD?

**Hypothesis 1:** Building on evidence that teachers’ role beliefs are context-sensitive and may shift across training and student-group contexts (Fives and Buehl, 2012; Vidović and Domović, 2019; Niikko, 2022), and drawing on metaphor-based work that organizes role beliefs into comparable orientations (Domović et al., 2017; Xin et al., 2020; Alaklabi and Low, 2022), we hypothesize that pre-service teachers’ distributions of role-belief orientations will differ systematically between typically developing students and students with IDD.

1. To what extent are pre-service teachers’ role beliefs about typically developing students associated with their beliefs about students with IDD?

**Hypothesis 2:** Guided by Rokeach’s (1968) Theory of Belief Systems, which suggests that earlier-formed, more psychologically embedded beliefs can provide interpretive frames that anchor subsequent beliefs (Pajares, 1992; Richardson, 2003; Gao and Cui, 2024), we hypothesize that pre-service teachers’ role-belief orientations toward typically developing students will be systematically associated with their role-belief orientations toward students with IDD. If observed, this pattern would be consistent with, but would not by itself prove, an anchoring relationship or the centrality of one belief set over another. These associations may persist after controlling for relevant background variables, such as academic year and program type.

## Methods

### Setting and participants

In China, “normal universities” are higher education institutions with a long tradition of teacher training. Although many have evolved into multidisciplinary universities, they remain central to the national teacher preparation system, are present in nearly every province, and are recognized for cultivating future educators. This study was conducted at a normal university in eastern China and involved pre-service teachers from both general education (i.e., kindergarten and primary school) and special education programs.

In the first and second years, the curricula of both programs emphasize foundational theoretical courses (e.g., Introduction to Education, General Psychology, Educational Ethics). In addition, general education pre-service teachers take specialized electives in special needs and inclusive education, typically delivered in large lecture classes of 50–70 students, to prepare them to teach students with IDD in mainstream classrooms. Special education pre-service teachers receive similar foundational coursework alongside more targeted training in inclusive and special education, usually in smaller classes of fewer than 30 students. In the third and fourth years, the curricula shift toward practice-oriented learning, integrating theory with hands-on training and internship experiences. Advanced coursework aligns with each program’s orientation: general education students focus on subject-specific teaching theories and practices, whereas special education students focus on the psychology, development, and education of students with IDD. Together, these programs aim to prepare high-quality educators capable of teaching in kindergartens, primary schools, and special education settings. They also seek to equip all pre-service teachers, including those in general education, with the knowledge and skills needed to support students with IDD.

Although the two programs differed in the intensity and specificity of their preparation for teaching students with IDD, they were situated within the same institutional teacher education context and shared a common foundation in general pedagogy, educational psychology, educational ethics, and inclusive education. This shared context was relevant to the present study because the research questions focused on how the same pre-service teachers construed their roles in relation to two target student groups, namely typically developing students and students with IDD, rather than on differences between teacher education programs. For this reason, data from participants in both programs were pooled and analyzed together in the main analyses. This analytic decision should not be interpreted as assuming that pre-service teachers in general and special education were empirically equivalent. Rather, it indicates that program differences were not the primary object of inquiry in the present study. To reduce the risk that program-specific variation would be overlooked, program type was also included as a covariate in the multinomial regression analyses.

Ethical approval for this study was obtained from the university’s Committee on Human Research Protection. Pre-service teachers in general and special education were recruited through convenience sampling, based on availability and willingness to participate. After obtaining informed consent, questionnaires were distributed in classrooms and dormitories. Participants could complete the questionnaire immediately or return it within 2 weeks. Of the 506 questionnaires distributed, 445 were returned, yielding a response rate of 87.94%. After excluding those with incomplete demographic information or unfinished metaphor tasks, 356 valid questionnaires remained, resulting in an effective response rate of 80.00%. Of the participants, 20 were male (5.62%) and 336 were female (94.38%). By academic year, 86 were first-year students (24.16%), 129 were second-year students (36.24%), 95 were third-year students (26.69%), and 46 were fourth-year students (12.92%). By program type, 289 were enrolled in general education programs (81.18%) and 67 in special education programs (18.82%).

### Instruments

A two-part questionnaire, adapted from previous metaphor research on teachers’ role beliefs (Domović et al., 2017; Xin et al., 2020), was used. The first part collected demographic information, including gender, academic year, and program. The second part comprised two semi-structured questions designed to elicit metaphorical responses and justifications. Metaphors were used to render abstract concepts familiar, thereby generating new conceptual insights (Gao and Cui, 2024; Ulusoy, 2022). Participants were given instructions and an example. For instance: “A doctor is like a shining angel of life under the sun, because they offer their love and warmth to every patient.” The two prompt questions were:

1. A teacher who teaches typically developing students is like ______ because ______.
2. A teacher who teaches students with IDD is like ______ because ______.

### Qualitative data coding

Content analysis, grounded in cognitive metaphor theory, was used to examine pre-service teachers’ role beliefs, drawing on Domović et al. (2017) and the Xin et al.’s (2020) work. In the first stage (familiarization), the first and second authors reviewed the raw data, repeatedly reading participants’ metaphorical responses. Key words and images (e.g., “bridge,” “parent,” “scaffolding”) were annotated to capture underlying beliefs. Reflective notes and discussions helped clarify emerging patterns, and personal assumptions were bracketed to ensure interpretations were grounded in participants’ perspectives.

In the second stage (preliminary coding), the metaphorical “vehicles” (e.g., “gardener” or “lighthouse”) were systematically extracted from participants’ responses. Each metaphor was coded separately for typically developing students and for students with IDD. The first and second authors also recorded participants’ justifications for their metaphors. For example, one participant described themselves as a “gardener” to foster each student’s potential over time, while another chose “lighthouse” to highlight the teacher’s role in guiding students through difficulties and uncertainties. These justifications provided critical insights into participants’ reasoning, revealing nuanced perceptions of professional responsibilities, instructional approaches, and the challenges of supporting diverse learners. This analysis laid the groundwork for subsequent thematic classification.

In the third stage (classification), metaphorical vehicles and their justifications were analyzed in depth. Each metaphor was interpreted in the context of its accompanying explanation. Metaphors were compared across participants to identify recurring patterns and themes. Through discussion and consensus, clear classification criteria were established, linking metaphorical meanings to broader categories of teachers’ role beliefs. All responses were systematically assigned to one of four orientations, namely behavioral, facilitative, protective, and self-centered. Table 1 presents the operational definition of each orientation, along with illustrative examples. These orientations served as analytic categories for interpreting participants’ metaphors and accompanying explanations, rather than as direct measures of fixed internal cognitive structures. In this sense, they capture recurring interpretive patterns in participants’ situated responses, which may reflect contextual and discursive influences, as well as features of the elicitation task itself.

**Table 1:** Metaphor-based analytic orientations used to interpret teachers’ role beliefs.

Orientations	Interpretive operational definition	Examples
Behavioral	The response was interpreted as emphasizing direct instruction and authority, prioritizing control over classroom activities and students’ learning outcomes rather than student autonomy.	A teacher who teaches typically developing students is like a mountain spring, because they smooth the jagged edges of rocks. A teacher who teaches students with IDD is like a trainer, because they continually refine children’s will and skills.
Facilitative	The response was interpreted as emphasizing support for students’ learning and autonomy, adopting strategies that enable students to participate actively, develop skills, and co-construct knowledge.	A teacher who teaches typically developing students is like a bright lamp, because they guide children toward a bright future. A teacher who teaches students with IDD is like a lighthouse in a storm, because they provide encouragement, support, and guidance.
Protective	The response was interpreted as emphasizing care and protection, thereby fostering a nurturing, emotionally safe, and supportive classroom.	A teacher who teaches typically developing students is like the sun, because they bring warmth and hope. A teacher who teaches students with IDD is like a gardener, because they carefully nurture students with special needs.
Self-centered	The response was interpreted as emphasizing the teacher’s efforts, performance, or expertise, with a focus on self-sacrifice, personal teaching identity, or skill demonstration rather than on students’ learning or needs.	A teacher who teaches typically developing students is like a candle, because they burn themselves to illuminate others. A teacher who teaches students with IDD is like a diligent gardener, because they selflessly cultivate each child with patience and perseverance.

In the fourth stage (inter-rater reliability), a pre-coding phase was conducted on a random sample of metaphorical responses from 46 pre-service teachers, covering role beliefs for both typically developing students and students with IDD. The first and second authors independently coded these metaphors using the established categories, carefully considering participants’ justifications. Inter-rater reliability was calculated using Miles and Huberman’s (1994) formula: Reliability = (Number of Agreements / (Number of Agreements + Number of Disagreements)) × 100%. Initial reliability scores were 84.86% for metaphors about typically developing students and 84.31% for those about students with IDD. Discrepancies were resolved through detailed discussions that examined the rationale for each coding decision and drew on participants’ explanations to reach consensus. The full dataset was then coded using the refined classification criteria.

During the formal coding phase, inter-rater reliability rose to 90.73% for metaphors about typically developing students and to 91.02% for those about students with IDD. Both figures exceeded the 90% threshold recommended by Miles and Huberman (1994), demonstrating the rigor and consistency of the coding process.

### Quantitative data analysis

SPSS 22.0 was used to analyze the distributions and patterns of metaphorical vehicles and role-belief orientations. Analyses tested the hypotheses corresponding to RQ1 and RQ2. For RQ1 (Hypothesis 1), the distributions of metaphorical vehicles across the two student groups were first compared descriptively. Chi-square tests of independence were then used to assess whether the distribution of teachers’ role-belief orientations differed between typically developing students and students with IDD. When the chi-square test was statistically significant, adjusted standardized residuals were examined for cell-wise *post hoc* inspection to identify which orientations were over- or under-represented in each group. Following common practice, cells with adjusted standardized residuals greater than 1.96 in absolute value were considered notable at approximately the 0.05 level (two-tailed).

For RQ2 (Hypothesis 2), the association between role-belief orientations for typically developing students and those for students with IDD was first examined using a chi-square test of independence, and Cramér’s *V* was reported to quantify the strength of the association. Adjusted standardized residuals were also inspected (using the same criterion as above) to identify the cells contributing most to the overall chi-square statistic. Multinomial logistic regression was then performed because the outcome variable (role-belief orientation for students with IDD) is nominal with more than two unordered categories. In these models, role-belief orientation for students with IDD was the dependent variable, role-belief orientation for typically developing students was the focal predictor, and gender, academic year, and program type were included as covariates. Program type was included to account for participants’ teacher education background while preserving the study’s primary focus on cross-target role-belief relations within the pooled sample. This strategy does not eliminate all program-specific differences, but it helps assess whether the observed associations remain when program background is statistically taken into account. The protective orientation was specified as the reference category for the outcome variable. For categorical predictors, one group was designated as the reference (e.g., female for gender, fourth-year for academic year, general education program) to facilitate interpretation of comparisons. Odds ratios (*ORs*) were used to interpret the results. An *OR* > 1 indicated higher odds of being in a given non-reference orientation (versus the reference category), whereas an *OR* < 1 indicated lower odds, holding all other predictors constant. All tests were two-tailed with *α* = 0.05.

## Results

### Comparison of pre-service teachers’ role beliefs for typically developing students and students with IDD (RQ1)

Table 2 presents the distribution of metaphorical vehicles used by pre-service teachers to describe their roles with typically developing students and students with IDD. “Gardener” was the most frequent metaphor for both groups, accounting for 53.93% of responses for typically developing students but only 19.10% for students with IDD. Other metaphors showed smaller shifts. “Lamp/Lighthouse” metaphors were slightly more frequent for students with IDD (6.74%) than for typically developing students (3.65%), whereas “Candle” metaphors decreased marginally from 6.74 to 5.06%. “Sun” and “Spring Rain” remained relatively stable in frequency, while less frequent metaphors (e.g., “Key,” “Large Tree,” “Mother”) showed only minor differences. The “Other” category increased sharply from 25.84% for typically developing students to 58.15% for students with IDD, reflecting a wider diversity of metaphorical expressions. This greater diversity may suggest that pre-service teachers’ conceptions of their professional roles are more heterogeneous and less settled in these responses when working with students with IDD.

**Table 2:** Distribution of metaphorical vehicles.

Teachers’ role beliefs for typically developing students	Frequency (*n*)	Percentage (%)	Teachers’ role beliefs for students with IDD	Frequency (*n*)	Percentage (%)
Gardener	192	53.93	Gardener	68	19.10
Candle	24	6.74	Lamp/Lighthouse	24	6.74
Lamp/Lighthouse	13	3.65	Candle	18	5.06
Sun	11	3.09	Sun	13	3.65
Spring rain	11	3.09	Spring Rain	11	3.09
Key	7	1.97	Large Tree	10	2.81
Large tree	6	1.69	Mother	5	1.40
Others	89	25.84	Others	207	58.15

Table 3 presents the distribution of pre-service teachers’ role-belief orientations for typically developing students and students with IDD across the four orientations. A chi-square test indicated significant differences in the distribution of role-belief orientations between the two student groups [*χ*^2^_(3)_ = 39.39, *p* < 0.001, Cramér’s *V* = 0.24], indicating a statistically significant but modest association. *Post hoc* cell-wise examinations of adjusted standardized residuals clarified where these differences occurred. Specifically, behavioral beliefs were significantly over-represented for typically developing students and under-represented for students with IDD (adjusted residuals = 6.20 and −6.20, respectively). In contrast, facilitative beliefs were under-represented for typically developing students but over-represented for students with IDD (adjusted residuals = −2.31 and 2.31). Self-centered beliefs showed a similar pattern, being slightly under-represented for typically developing students and over-represented for students with IDD (adjusted residuals = −1.98 and 1.98). Protective beliefs did not differ significantly between groups, as indicated by adjusted residuals of −1.56 and 1.56, suggesting relative stability in this orientation across student groups.

**Table 3:** Comparison of teachers’ role belief orientation distributions for typically developing students and students with IDD.

Belief orientations	Teachers’ role beliefs for typically developing students *n* (%)	Teachers’ role beliefs for students with IDD *n* (%)	Total *n* (%)
Self-centered	68(19.10)	90(25.28)	158(22.19)
Behavioral	107(30.06)	40(11.24)	147(20.65)
Protective	120(33.71)	140(39.33)	260(36.52)
Facilitative	61(17.13)	86(24.16)	147(20.65)

To further interpret these residual-based differences, the relative prominence of each orientation across the student groups was examined. Distinct patterns emerged across the four orientations. Protection-oriented beliefs were the most prevalent in both groups and comparatively stable (33.71% for typically developing students vs. 39.33% for students with IDD), consistent with the non-significant residuals for this orientation. This orientation was expressed in caring terms, with teachers described as “caretakers” who “extend their arms to protect every child” and “warm students with unconditional love.” Behavioral beliefs were markedly more prevalent for typically developing students than for students with IDD (30.06% vs. 11.24%), framing teachers as “shapers” or “transmitters,” exemplified by the view that “only through a gardener’s pruning can flowers become more beautiful.” In contrast, facilitative beliefs accounted for a larger share when participants considered students with IDD than when they considered typically developing students (24.16% vs. 17.13%), pointing to a greater emphasis on individualized guidance and adaptive instruction. Participants often framed this role in supportive terms, depicting teachers as “guides” who “facilitate students’ progress.” Similarly, self-centered beliefs constituted a larger share for students with IDD than for typically developing students (25.28% vs. 19.10%), suggesting heightened perceived personal effort and emotional investment in inclusive contexts. In these accounts, teachers were characterized as “dedicated workers” who “pour their hearts and souls into their efforts.”

Overall, the observed differences in orientation distributions between typically developing students and students with IDD were consistent with Hypothesis 1. Behavioral beliefs were more prevalent for typically developing students, facilitative and self-centered beliefs accounted for a somewhat larger share among students with IDD, and protective beliefs remained comparatively stable across groups.

### Associations between pre-service teachers’ role beliefs for typically developing students and students with IDD (RQ2)

Table 4 presents associations between pre-service teachers’ role-belief orientations for typically developing students and for students with IDD. A chi-square test of independence indicated a significant association between the two sets of orientations [*χ*^2^(9) = 35.07, *p* < 0.001, Cramér’s *V* = 0.18], reflecting a statistically significant but modest association. Adjusted standardized residuals further clarified which cells contributed most to this association. Across orientations, continuity was most evident along the diagonal. Teachers who expressed self-centered role beliefs for typically developing students most often reported the same orientation for students with IDD (44.12%), and this self-centered-to-self-centered cell was over-represented relative to independence expectations (adjusted residual = 3.97). Behavioral (19.63%; adjusted residual = 3.29), protective (47.50%; adjusted residual = 2.25), and facilitative (36.07%; adjusted residual = 2.39) beliefs showed similar within-orientation continuity, with each corresponding diagonal cell contributing positively to the overall association. At the same time, the cross-tabulation revealed substantively meaningful cross-orientation shifts. For example, among teachers with behavioral beliefs for typically developing students, sizeable proportions endorsed protective (39.25%) or facilitative (21.50%) beliefs for students with IDD, suggesting a tendency to move from behavioral control toward care and instructional support when considering students with IDD.

**Table 4:** Association between teachers’ role belief orientation for typically developing students and students with IDD.

Belief orientations		Teachers’ role beliefs for typically developing students				
		Self-centered *n* (%)	Behavioral *n* (%)	Protective *n* (%)	Facilitative *n* (%)	Total *n* (%)
Teachers’ role beliefs for students with IDD	Self-centered	30(44.12)	21(19.63)	27(22.50)	12(19.67)	90(25.28)
	Behavioral	6(8.82)	21(19.63)	5(4.17)	8(13.11)	40(11.24)
	Protective	22(32.25)	42(39.25)	57(47.50)	19(31.15)	140(39.33)
	Facilitative	10(14.71)	23(21.50)	31(25.83)	22(36.07)	86(24.16)

Further analysis used multinomial logistic regression to assess whether gender, academic year, program type, and teachers’ role beliefs regarding typically developing students were associated with pre-service teachers’ role-belief orientations for students with IDD. In this model, the protective orientation was set as the reference outcome category (see Table 5). The reference groups for the predictors were female (gender), fourth-year (academic year), general education (program type), and the protective orientation (for typically developing students’ role beliefs). The omnibus likelihood-ratio test indicated that the full model fit the data better than the intercept-only model [*χ*^2^_(24)_ = 81.50, *p* < 0.001]. Results were interpreted using ORs comparing each non-reference outcome with the protective outcome, with other predictors held constant.

**Table 5:** The multinomial logistic regression model of beliefs about teacher roles for students with IDD.

Variables	Self-centered beliefs about teacher roles for students with IDD (Protective orientation as the reference group)							Behavioral beliefs about teachers roles for students with IDD (Protective orientation as the reference group)							Facilitative beliefs about teacher roles for students with IDD (Protective orientation as the reference group)						
	*B*	*SE*	Wald	*p*	OR	95%*CI*		*B*	*SE*	Wald	*p*	OR	95%*CI*		*B*	*SE*	Wald	*p* LL	OR	95%*CI*	
						LL	UL						LL	UL						LL	UL
Gender (Female as the reference group)																					
Male	−0.07	0.68	0.01	0.92	0.93	0.25	3.53	1.25	0.66	3.57	0.06	3.49	0.95	12.76	0.12	0.69	0.03	0.87	1.12	0.29	4.32
Academic year (Fourth year as the reference group)																					
First year	1.82	0.63	8.40	0.004	6.15	1.80	21.01	0.88	0.73	1.44	0.23	2.40	0.57	10.08	−0.36	0.46	0.60	0.44	0.70	0.28	1.73
Second year	1.14	0.61	3.49	0.06	3.13	0.95	10.38	−0.58	0.77	0.56	0.45	0.56	0.12	2.53	−0.20	0.41	0.24	0.62	0.82	0.37	1.83
Third year	1.68	0.62	7.44	0.006	5.36	1.60	17.92	0.40	0.73	0.30	0.58	1.49	0.36	6.23	−1.26	0.51	6.15	0.01	0.28	0.11	0.77
Program type (General education as the reference group)																					
Special education	−0.09	0.38	0.05	0.82	0.92	0.43	1.94	0.08	0.51	0.03	0.87	1.09	0.40	2.96	−0.59	0.36	2.73	0.10	0.56	0.28	1.12
Teachers’ role beliefs for typically developing students (Protective orientation as the reference group)																					
Self-centered	1.06	0.38	7.88	0.005	2.89	1.38	6.05	1.14	0.67	2.93	0.09	3.13	0.85	11.54	−0.14	0.45	0.10	0.75	0.87	0.36	2.09
Behavioral	−0.11	0.36	0.09	0.77	0.90	0.44	1.83	1.65	0.56	8.82	0.003	5.20	1.75	15.44	0.16	0.36	0.19	0.66	1.17	0.58	2.36
Facilitative	0.14	0.45	0.10	0.75	1.15	0.48	2.77	1.52	0.65	5.54	0.02	4.58	1.29	16.27	0.92	0.40	5.27	0.02	2.51	1.14	5.50

For the self-centered (vs. protective) outcome, first-year (*OR* = 6.15, 95% *CI* [1.80, 21.01], *p* = 0.004) and third-year (*OR* = 5.36, 95% *CI* [1.60, 17.92], *p* = 0.006) students had higher odds than fourth-year students of endorsing self-centered rather than protective role beliefs for students with IDD. Additionally, self-centered role beliefs for typically developing students (vs. protective) were associated with higher odds of endorsing self-centered (vs. protective) beliefs for students with IDD (*OR* = 2.89, 95% *CI* [1.38, 6.05], *p* = 0.005). For example, this pattern is reflected in paired metaphors such as describing a teacher of typically developing students as “a candle, because they wear themselves down to light every student’s path,” and a teacher of students with IDD as “a watchmaker, because they must rely on extraordinary patience, endurance, and skill in every tiny adjustment.” In both cases, the emphasis remained on the teacher’s own effort and professional performance.

For the behavioral (vs. protective) outcome, endorsing behavioral (*OR* = 5.20, 95% *CI* [1.75, 15.44], *p* = 0.003) or facilitative (*OR* = 4.58, 95% *CI* [1.29, 16.27], *p* = 0.02) role beliefs for typically developing students (each vs. protective) was associated with higher odds of endorsing behavioral (vs. protective) beliefs for students with IDD. One kind of continuity appeared in paired metaphors, such as describing a teacher of typically developing students as “a captain, because they set the rules and keep the class on course,” and a teacher of students with IDD as “a drill coach, because they break learning into fixed steps and use repeated practice until each action is performed correctly.” Another pattern involved a shift from facilitative to behavioral beliefs, as when a teacher of typically developing students was described as “a bridge, because they connect students to new ideas and help them move toward independent learning,” but a teacher of students with IDD was described as “a driving instructor, because they direct each step, correct each action, and use repetition until routines become stable.” These examples suggest that, in the IDD context, support was often reinterpreted as structure, correction, and routine formation.

For the facilitative (vs. protective) outcome, third-year students had lower odds than fourth-year students (OR = 0.28, 95% *CI* [0.11, 0.77], *p* = 0.01). However, endorsing facilitative role beliefs for typically developing students (vs. protective) was associated with higher odds of endorsing facilitative (vs. protective) beliefs for students with IDD (*OR* = 2.51, 95% *CI* [1.14, 5.50], *p* = 0.02). This continuity is evident in paired metaphors, such as describing a teacher of typically developing students as “a bridge, because they connect students to new ideas and help them move toward independent thinking,” and a teacher of students with IDD as “a scaffold, because they provide adapted support for participation, skill development, and gradual independence.” In both cases, support remained student-centered rather than shifting toward control.

Taken together, the cross-tabulation and multinomial regression results supported Hypothesis 2 by showing systematic associations between teachers’ role-belief orientations for typically developing students and for students with IDD. These findings suggest both continuity within the same orientation and selective links between orientations, even after controlling for background variables.

## Discussion

This study provides evidence that pre-service teachers’ role beliefs about typically developing students and those about students with IDD are systematically associated. These associations may reflect interconnected patterns within emerging professional belief systems. For RQ1, we found clear parallels and distinctions in role-belief orientations between the two student groups. Behavioral orientations were more prevalent for typically developing students, facilitative and self-centered orientations were more prominent for students with IDD, and protective orientations remained comparatively stable across groups. For RQ2, we found that role-belief orientations for typically developing students were systematically associated with those for students with IDD. These patterns are consistent with a possible anchoring interpretation, although they should not be taken as direct evidence of belief centrality, hierarchical organization, or a fully specified anchoring mechanism.

Building on these findings, this study makes three key contributions to research on teacher belief systems and inclusive teacher education. First, on the methodological front, it advances metaphor-based research by integrating protocol-based coding with a within-participant cross-group design. This design directly compares role beliefs for typically developing students and students with IDD and models their interrelations, moving beyond prior metaphor studies that often focused on a single student population or failed to examine how role beliefs across student groups are linked within the same individuals (Xin et al., 2020; Domović et al., 2017). Importantly, by applying this approach to students with IDD in the Chinese teacher education context, the study addresses a documented gap in subgroup-specific evidence and provides contextually grounded insights into belief formation under contemporary inclusion policies and training realities. Second, empirically, it delineates distinct patterns in role beliefs across student groups, revealing that protective orientations were comparatively stable and prominent, while instructional orientations diverged between typically developing students and students with IDD. These nuanced findings inform, rather than determine, the design of targeted interventions in inclusive teacher education. Third, theoretically, these results offer tentative support for Rokeach’s (1968) account of belief systems, as they suggest a pattern that may be compatible with an anchoring interpretation. In this process, role beliefs for typically developing students may function as reference frames, constraining how teachers construe their roles for students with IDD, while still allowing for variability and malleability. Such an interpretation remains provisional because the present study does not directly measure the centrality or organization of beliefs. In doing so, this study supports a cautious interpretation that teachers’ metaphorically expressed role beliefs may reflect dynamic and interconnected ways of construing teaching roles across student groups, rather than a static set of attitudes (Fives and Buehl, 2012).

### Stable protective role belief orientations, but divergent behavioral, self-centered, and facilitative orientations across student groups

Regarding similarities, protective orientations predominated among Chinese pre-service teachers in both student groups. This pattern aligns with prior international research, underscoring the cross-contextual recurrence of protective beliefs (Alaklabi and Low, 2022; Vidović and Domović, 2019). Protective beliefs may be a particularly salient and recurrent element of teachers’ professional belief systems, shaped by deeply ingrained moral and socialization norms in teacher training and early schooling. These norms emphasize care, empathy, and student welfare. In China, this pattern aligns with the longstanding professional culture that prioritizes “caring for students” and moral education, with teacher education programs explicitly fostering responsibility and attentiveness through courses such as Educational Psychology and Educational Ethics. Consequently, Chinese pre-service teachers’ strong endorsement of protective roles across both typically developing students and students with IDD reflects the internalization of these core values, suggesting that protective role framings may be highly salient across student groups, although their psychological centrality was not directly measured in this study.

Despite this shared core, protective beliefs were endorsed more strongly for students with IDD than for typically developing students. This finding extends prior research, which typically examined role beliefs for either typically developing students (Vidović and Domović, 2019) or students with disabilities (Alaklabi and Low, 2022), by comparing multiple student groups simultaneously and highlighting the differential emphasis placed on learners with diverse needs. The stronger endorsement for students with IDD likely reflects teachers’ heightened perception of vulnerability, emphasizing care and safeguarding (Domović et al., 2017). It also indicates that, while protective orientations appeared salient in both groups in the present study, their relative emphasis varied with student characteristics, illustrating how potentially important beliefs interact with the perceived needs of different learner groups.

Pre-service teachers’ role beliefs diverged markedly: those for typically developing students were predominantly behavioral, whereas those for students with IDD were more often self-centered or facilitative. This distinction is notable, as few studies have systematically examined how pre-service teachers differentiate their role beliefs across student groups, particularly in the Chinese context (Xin et al., 2020). It suggests that more familiar or pre-existing role beliefs about typically developing students may be associated with, and potentially frame, role beliefs about students with IDD. The predominance of behavioral beliefs for typically developing students aligns with Ulusoy’s (2022) longitudinal study in Turkey, which found that primary school teacher education participants consistently generated teacher-centered, behaviorist metaphors throughout their pre-service and early in-service years. This pattern reflects deeply ingrained beliefs reinforced by conventional teacher education. Similarly, Chinese pre-service teachers rely on established pedagogical models and culturally transmitted norms that emphasize measurable academic outcomes, classroom management, and orderly learning environments (Lo, 2021). In China, foundational teacher training prioritizes classroom management, knowledge transmission, and standardized instruction within an exam-oriented, discipline-focused culture, reinforcing behavioral orientations for typically developing students (Wang and Yang, 2021). This pattern illustrates how professional preparation and broader sociocultural norms jointly shape role beliefs, highlighting the importance of targeted pedagogical interventions to promote more student-centered and inclusive practices (Domović et al., 2017).

The higher prevalence of self-centered and facilitative beliefs about students with IDD aligns with prior domestic research. Chinese pre-service special education teachers primarily endorse facilitative and self-centered orientations toward students with disabilities (Xin et al., 2020), and a similar pattern has been observed among STEM-oriented pre-service teachers working with migrant students (Wang and Yang, 2021).

At the same time, interpreting self-centered orientations requires conceptual differentiation. Self-centered orientations may reflect pre-service teachers’ recognition that supporting students with IDD requires substantial personal effort, dedication, and moral responsibility. In China, teachers are socialized to value diligence, perseverance, and accountability, and pre-service training emphasizes personal responsibility for student outcomes (Lo, 2021). As a result, pre-service teachers may perceive a heightened ethical and professional obligation to invest fully in students’ learning and development, reinforcing self-centered beliefs as a salient aspect of their professional role conception (Xin et al., 2020). This form of self-investment can be productive when it supports persistence, professional commitment, and sustained instructional care. However, self-centered beliefs may also be limiting if they shift toward a teacher-sacrifice narrative that positions students as passive recipients and inadvertently constrains student agency. Accordingly, we view self-centered beliefs as potentially adaptive yet context-dependent—and in some cases transitional—with their implications for inclusive practice contingent on whether teachers’ self-investment is coupled with autonomy-supportive expectations and shared responsibility within a broader support system.

Facilitative orientations, by contrast, emphasize individualized attention, differentiated instruction, and adaptive strategies tailored to students’ unique learning needs. Recent advances in inclusive education in China have heightened pre-service teachers’ awareness of the importance of personalized support, fostering facilitative beliefs that reflect emerging commitments to equity and inclusive practices (Fu, 2022). Together, these patterns suggest that teacher education should not only cultivate facilitative approaches but also guide pre-service teachers in translating their self-investment into practices that strengthen—rather than replace—students’ participation and agency.

The divergence in pre-service teachers’ role beliefs about typically developing students and those with IDD can be explained by differences in educational experiences and cultural expectations. Extensive practicum and coursework with typically developing students—which emphasize instruction, classroom management, and maintaining order—reinforce behavioral role beliefs that prioritize structured teaching and oversight (Domović et al., 2017; Ulusoy, 2022). In contrast, limited experience with students with IDD encourages interpretations of professional roles centered on moral responsibility, care, and individualized support, fostering self-centered and facilitative orientations. Cultural and systemic expectations further contribute to these differences: mainstream education in China emphasizes academic achievement and classroom discipline, whereas special and inclusive education encourages teachers to act as supportive guides attentive to students’ diverse learning needs (Yu et al., 2011).

### Academic year and teachers’ role beliefs for typically developing students are associated with role beliefs for students with IDD

The observed developmental differences suggest that pre-service teachers’ role beliefs evolve throughout their training. In the early stages of teacher preparation, first- and third-year pre-service teachers were more likely than fourth-year students to hold self-centered beliefs (with the protective orientation as the reference category). This indicates that early in training, their role beliefs remain relatively unstable and inward-focused, emphasizing personal effort over students’ needs. By the fourth year, however, the likelihood of adopting a facilitative orientation increased compared with that of third-year students, reflecting the cumulative influence of professional coursework and an extended, immersive teaching practicum. These findings suggest a developmental trend in which role beliefs shift from predominantly self-centered toward more facilitative orientations, which may indicate maturation in professional understanding and the gradual formation of inclusive teaching conceptions. A similar trajectory was documented in Vidović and Domović’s (2019) longitudinal study of Croatian pre-service teachers, which showed early protective orientations giving way to facilitative, student-centered beliefs through practical experience and reflection. In China, this pattern aligns with the structure of teacher education programs: the early years (first through third) emphasize theoretical training, core pedagogical knowledge, and ethical responsibilities, while the fourth year provides sustained, hands-on opportunities to apply inclusive education principles and adopt student-centered approaches (Xin et al., 2020).

Previous studies have examined how teachers’ broader belief systems relate to teaching specific student groups. These works have either emphasized the impact of beliefs about students with disabilities on teachers’ roles or explored how general role beliefs influence teaching, learning, and inclusion (Alaklabi and Low, 2022). In contrast, our study indicates that pre-service teachers’ role beliefs about typically developing students are systematically associated with their beliefs about students with IDD. This finding is consistent with Rokeach’s (1968) Theory of Belief Systems, which suggests that earlier-formed, more psychologically embedded beliefs may provide interpretive frames for subsequent belief development. However, because the present study did not directly measure belief centrality, interconnectedness, or resistance to change, the findings should be interpreted as supporting a possible anchoring reading rather than demonstrating a hierarchical belief structure. Rather than confirming the framework, our results provide a context-specific empirical pattern that can be interpreted through it, in which more familiar role beliefs about typically developing students may serve as reference frames for beliefs about students with IDD. This underscores the possible stability of pre-existing role beliefs alongside their context-sensitive flexibility. In doing so, our study makes a more modest theoretical contribution by suggesting how pre-existing role beliefs about typically developing students may shape or channel the development of inclusive teaching conceptions for students with IDD.

Self-centered role beliefs about typically developing students were generally maintained by pre-service teachers when considering students with IDD, a pattern that may be compatible with the persistence of early-formed beliefs emphasizing effort, responsibility, and dedication (Wang and Yang, 2021). In China, such orientations are reinforced by cultural and institutional norms that value diligence and moral responsibility, as well as by teacher education programs that emphasize personal commitment to student outcomes (Wang, 2023). From a belief-system perspective (Pajares, 1992; Rokeach, 1968), these self-centered beliefs may function as one possible interpretive frame that guides pre-service teachers’ understanding of their responsibilities across student groups. This may help explain part of their continuity across contexts, including when pre-service teachers consider students with IDD.

Facilitative role beliefs about typically developing students are also associated with beliefs about students with IDD, suggesting that established facilitative orientations may provide a framework for supporting engagement, autonomy, and learning (Alaklabi and Low, 2022). In China, teacher education programs cultivate these beliefs through inclusive education coursework, structured practicum experiences, and reflective activities, encouraging the adoption of student-centered approaches (Lo, 2021). From a belief-systems perspective (Pajares, 1992; Rokeach, 1968), facilitative beliefs may serve as a reference framework that shapes teachers’ role beliefs across student populations, contributing to the continuity observed between beliefs about typically developing students and those about students with IDD. This continuity is particularly encouraging, as it indicates that pre-service teachers can retain or transfer student-centered orientations to inclusive contexts, forming a foundation for effective inclusive classroom practices (Fu, 2022; Wang, 2023). Together, the patterns observed for self-centered and facilitative beliefs highlight how pre-existing, potentially well-connected beliefs may contribute to continuity in teacher cognition and support adaptive, inclusive teaching, offering a possible insight into how belief systems guide approaches to diverse learners.

A similar pattern was observed for behavioral role beliefs: pre-service teachers who endorsed behavioral beliefs for typically developing students were also more likely to endorse them for students with IDD. Notably, behavioral beliefs about students with IDD were predicted not only by behavioral beliefs for typically developing students but also by facilitative beliefs for typically developing students. This pattern may seem counterintuitive, but it should be interpreted cautiously. Because the multinomial regression used the protective orientation as the reference outcome, the result indicates that facilitative beliefs for typically developing students were associated with higher odds of behavioral rather than protective beliefs for students with IDD; it does not directly show that behavioral beliefs were more likely than facilitative beliefs. Indeed, facilitative beliefs for typically developing students were also associated with higher odds of facilitative rather than protective beliefs for students with IDD. Taken together, these findings suggest that facilitative beliefs in typical-student contexts may be linked to more than one IDD-related orientation, rather than mapping onto a single, uniform pattern.

To clarify how this unexpected association might arise, we revisited the qualitative data and examined paired metaphorical responses from participants whose metaphors were coded as facilitative for typically developing students but behavioral for students with IDD. This follow-up analysis suggested that some participants did not abandon facilitative ideals when considering students with IDD; rather, they appeared to reinterpret those ideals through more structured, directive forms of support. For instance, some participants used facilitative metaphors for typically developing students that emphasized guidance, openness, and student agency, portraying the teacher as supporting exploration while allowing students to find their own direction. However, when describing teachers of students with IDD, these same participants sometimes shifted to more directive metaphors that emphasized breaking tasks into small steps, establishing routines, and using repetition to stabilize appropriate responses. This pattern suggests that what is expressed as facilitation in one context may be recast as structured behavioral support in another, particularly when students are perceived as needing more explicit guidance. Thus, facilitative ideals may, in some cases, be reconfigured as behavioral structure, repeated practice, correction, or habit formation in IDD-related contexts.

Accordingly, we interpret this finding as a possible indication of structured-support reasoning, hybrid role beliefs, or a limited IDD-specific pedagogical repertoire. Pre-service teachers may endorse facilitative principles in general, but when they perceive students with IDD as requiring more intensive instructional support, familiar behavioral scripts may become more salient (Li et al., 2024; Ulusoy, 2022; Wang and Yang, 2021). From this perspective, the apparent shift from facilitative to behavioral orientations may reflect not a simple contradiction, but a less differentiated understanding of how student-centered support should be enacted in IDD-related contexts. This finding points to the value of helping pre-service teachers translate general facilitative beliefs into concrete, autonomy-supportive, and evidence-informed strategies for students with IDD, rather than relying mainly on corrective or control-oriented role scripts in uncertain conditions (Alaklabi and Low, 2022; Domović et al., 2017).

### Implications for teacher education

These findings have important implications for cultivating adaptive, inclusive role beliefs among pre-service teachers. First, salient protective beliefs should be leveraged to support constructive, inclusive practices rather than reinforcing overprotection or low expectations for students with IDD. Second, the observed divergence between behavioral orientations for typically developing students and facilitative orientations for students with IDD underscores the need to help pre-service teachers flexibly adjust their role enactments and actively develop facilitative beliefs toward students with IDD. Relying solely on existing behavioral beliefs is insufficient; targeted interventions are needed to foster genuinely student-centered approaches. Teacher education programs should provide structured opportunities to practice student-centered strategies, implement differentiated instruction, and engage in guided reflection on inclusive teaching. Extended field experiences with students with IDD, supported by mentoring and feedback from experienced inclusive educators, can reinforce facilitative orientations and help teachers transition from controlling or purely behavioral approaches to strategies that promote engagement, autonomy, and learning. Third, because beliefs about typically developing students and students with IDD can become conflated, programs should explicitly address both domains. Structured reflection and inclusive field experiences can help pre-service teachers integrate their beliefs, manage general classrooms effectively, and provide targeted support for students with IDD, thereby advancing inclusive education in China.

Moreover, the finding that beliefs about teacher roles for typically developing students were associated with beliefs about roles for students with IDD suggests that more familiar or pre-existing role beliefs may serve as possible anchors. Teacher education programs should explicitly engage pre-service teachers’ existing beliefs about typically developing students, helping them recognize how these pre-existing beliefs may shape perceptions and practices with students with IDD. Understanding this possible distinction between earlier-formed role beliefs and later-developing, context-specific role beliefs allows educators to guide belief development strategically. Case discussions can support pre-service teachers in comparing the learning needs of different student groups, identifying which core beliefs can be adaptively extended to students with IDD, and pinpointing beliefs that may lead to low expectations, enabling targeted adjustments. Additionally, the observed developmental patterns—first- and third-year pre-service teachers tending toward self-centered orientations, and fourth-year pre-service teachers increasingly emphasizing facilitative orientations—underscore the importance of sequencing theoretical and practical components in teacher education to align with these trajectories. By providing targeted courses, structured field experiences, and guided reflection, programs can engage pre-existing role beliefs to cultivate adaptive, student-centered practices, guiding pre-service teachers from self-focused orientations toward flexible, responsive approaches that meet the diverse needs of all students, including those with IDD, thereby potentially supporting inclusive teaching orientations.

### Limitations and future research

Several limitations should be noted. First, although the study included pre-service teachers from general and special education programs at a normal university in eastern China, the single-institution sample may limit the generalizability of the findings to other regions, school types, or training contexts. In addition, because participants from general and special education programs were pooled for the main analyses, program-specific nuances may have been obscured, even though program type was statistically controlled in the regression models. Future studies could recruit pre-service teachers from multiple institutions with varying program structures and curricula, including differences in general versus special education training, course emphasis, and practicum arrangements, to test whether the observed patterns hold across diverse teacher education contexts.

Second, the sample was predominantly female, which may have influenced the overall distribution of teachers’ role-belief orientations—particularly those emphasizing care, protection, and relational responsibility. Although gender was not a significant predictor in the multinomial regression models, future studies with more gender-balanced samples should examine whether gendered socialization or differential training experiences are associated with caring- or protective-role belief patterns.

Third, this study relied on responses to semi-structured questions for metaphor analysis, which may capture only part of the complexity of teachers’ beliefs. Metaphorical expressions are interpretive and context-dependent. They may reflect not only participants’ professional understandings but also culturally available discourses, the framing of the prompt, and immediate communicative choices. Accordingly, the identified orientations should be interpreted as analytic patterns rather than as direct representations of fixed internal cognitive structures. Future research could incorporate additional data sources, such as questionnaires, written reflections, or in-depth interviews, to more comprehensively examine the formation and dynamics of role beliefs.

Finally, the cross-sectional design precludes causal inference. Although beliefs about roles for typically developing students were associated with those for students with IDD, longitudinal studies are needed to track developmental trajectories and to examine how teacher education experiences shape the evolution of beliefs in both groups.

## Data Availability

The raw data supporting the conclusions of this article will be made available by the authors, without undue reservation.
